# Longitudinal pharmacokinetic and safety studies for dose optimization of a brain- penetrating erythropoietin for Alzheimer’s disease

**DOI:** 10.21203/rs.3.rs-6874797/v1

**Published:** 2025-06-17

**Authors:** Rudy Chang, Devaraj V. Chandrashekar, G. Chuli Roules, Nataraj Jagadeesan, Emi Iwasaki, Adenike Oyegbesan, Hayk Davtyan, Rachita K. Sumbria

**Affiliations:** Chapman University; Chapman University; Chapman University; Chapman University; Chapman University; Chapman University; University of California; Chapman University

**Keywords:** Pharmacokinetics, blood-brain barrier, erythropoietin, amyloid-beta, Alzheimer’s disease, transferrin receptor antibody, neuroprotection, APPSAA KI mice

## Abstract

**Background:**

Erythropoietin (EPO) is a potential therapeutic for Alzheimer’s disease (AD), but has limited brain penetration, requiring high systemic doses that lead to hematopoietic side effects. To overcome this, EPO was conjugated with a transferrin receptor monoclonal antibody (TfRMAb) to enhance blood-brain barrier transport. This study assessed the pharmacokinetics (PK), safety, and efficacy of this modified EPO after repeated dosing in mice.

**Methods:**

For the PK and safety study, a multidose design was employed with 10-week-old C57 male mice (n=4–5/dose) receiving low (1 mg/kg), mid (3 and 6 mg/kg), or high (20 mg/kg) doses SQ for 4 weeks, aimed to evaluate the dose-dependent plasma concentrations and biodistribution, and metabolic and hematologic safety of the modified EPO. The dose that resulted in the highest safety and sustained plasma exposure was then dosed SQ to 5.5-month-old male APP_SAA_ KI mice (n=6) for 14 weeks. Controls included vehicle-treated APP_SAA_ KI and APP wild-type mice (n=4–5/group). The effect of modified EPO on Aβ load by immunoassays and spatial memory via the Y-maze test were assessed.

**Results:**

The 1 mg/kg dose showed no adverse effects and sustained brain and plasma exposure following repeated dosing, making it suitable for longitudinal treatment. Mid and high doses reduced plasma and brain exposure, and altered hematocrit, TfR expression, and spleen weight; changes that were largely reversible upon treatment cessation. Reduced plasma exposure at mid and high doses was not completely explained by increased TfR expression, anti-drug antibodies, tissue sequestration, or EPO receptor expression. Subsequently, 5.5-month-old APP_SAA_ KI mice received 1 mg/kg TfRMAb-EPO SQ for 14 weeks. Modified EPO significantly reduced brain Aβ load (70–80%, p<0.001) and aggregated Aβ (p<0.05), and improved spatial memory, indicated by a higher discrimination index in the Y maze test (p<0.05).

**Conclusions:**

With the advancement of TfRMAb-based therapeutics into clinical trials for AD, these findings are particularly significant. They offer essential preclinical data to guide dose optimization in longitudinal studies using TfRMAb-based therapeutics, specifically modified-EPO, and show the robust therapeutic potential of low-dose brain-penetrating EPO in the APP_SAA_ KI AD mouse model.

## Introduction

Alzheimer’s disease (AD) is a progressive neurodegenerative disorder marked by hallmark pathologies such as amyloid-beta (Aβ) plaques, tau neurofibrillary tangles, neuroinflammation, and widespread neuronal loss [1]. While recent advances have led to the development of promising disease-modifying therapies, ongoing research is essential to fully establish their long-term safety and efficacy [2]. There remains a critical need for therapeutic strategies that not only target the underlying pathological features of AD but also offer robust neuroprotection—preserving cognitive function and promoting neuronal survival throughout disease progression.

Erythropoietin (EPO), a glycoprotein hormone traditionally associated with erythropoiesis, has shown promise as a neuroprotective agent in preclinical models of neurodegenerative diseases [3]. EPO exerts anti-apoptotic, anti-inflammatory, and pro-regenerative effects on neuronal cells, making it an attractive candidate for AD therapy [3]. However, its therapeutic potential is hindered by limited uptake across the blood-brain barrier (BBB) and hematopoietic effects associated with chronic high-dose systemic administration [4–6]. To overcome these limitations, EPO was re-engineered to bypass the BBB using a transferrin receptor monoclonal antibody (TfRMAb) [7], a strategy that facilitates receptor-mediated transcytosis into the brain parenchyma while minimizing peripheral adverse effects associated with high-dose chronic EPO dosing [8]. Notably, this approach is currently in a Phase 2 clinical trial for the brain delivery of an anti-amyloid antibody for AD (NCT04639050).

Our prior proof-of-concept work showed that this brain-penetrable modified EPO reduced AD hallmarks (Aβ and phosphorylated tau) in AD mouse models [8, 9]. However, the impact of longitudinal administration of TfRMAb-EPO on plasma pharmacokinetics (PK), tissue biodistribution, and systemic effects at different doses is unknown. In this study, we aimed to characterize the safety profile and PK of the modified EPO (TfRMAb-EPO) following chronic administration in healthy mice. This preclinical assessment enabled the determination of an optimized dosing regimen that was subsequently applied in an AD mouse model. Specifically, we evaluated the therapeutic efficacy of TfRMAb-EPO in APP_SAA_ knock-in (KI) mice, a transgenic model that recapitulates Aβ pathology without the confounding effects of human amyloid precursor protein (APP) overexpression [10]. By investigating the ability of TfRMAb-EPO to mitigate hallmark AD pathologies *in vivo*, this work provides insights into the feasibility of using modified EPO as a therapeutic approach for AD.

## Materials & Methods

### Pharmacokinetics and Safety Study

The TfRMAb-EPO fusion protein was produced via transient expression in Chinese hamster ovary (CHO-K1) cells and purified using protein A affinity chromatography (WuXi Biologics, SH, China) [7]. Based on amino acid sequence, the EPO domain comprises 20% of the TfRMAb-EPO fusion protein.

All animal procedures were approved by the Chapman University Institutional Animal Care and Use Committee (IACUC) and were carried out in compliance with university regulations. All the animals were housed in a temperature and humidity-controlled animal care facility under a 12-hour light-dark cycle, with *ad libitum* access to feed and drinking water. All animals were carefully observed after each injection for any signs of immune reactions, monitored daily for any immune reactions and behavioral changes, and body weights were recorded every week until the end of treatment [11].

**Figure 1:**
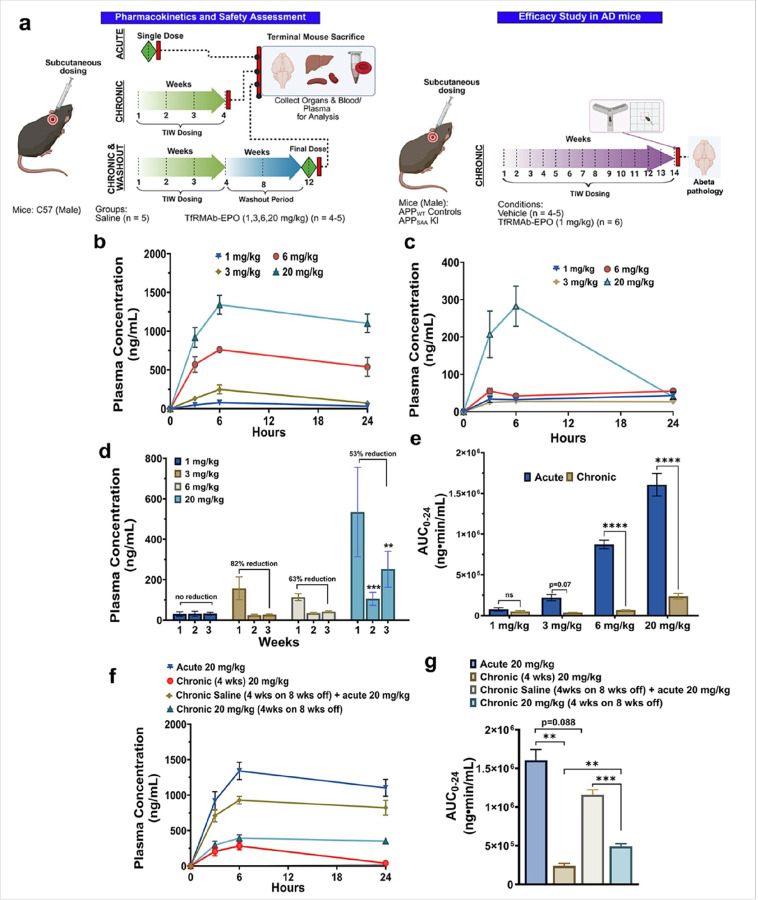
Plasma exposure of TfRMAb-EPO after acute and chronic dosing. Schematic view of the experimental design (a). Plasma concentration-time curves following acute (b) and chronic (c) dosing of TfRMAb-EPO. Weekly plasma TfRMAb-EPO concentrations after repeated dosing (d). Mice treated with 1 mg/kg of TfRMAb-EPO did not show a significant reduction in plasma concentration following 4 weeks of chronic dosing (d). Comparison of area under the plasma concentration-time curve (AUC_0–24_) following acute and chronic dosing (e). Plasma concentrations (f) and AUC_0–24_ (g) following a 20 mg/kg acute, chronic, and an eight-week washout, followed by a final 20 mg/kg dose. Data are represented as mean ± SEM of n = 4–6 per group and were analyzed using the one-way ANOVA with Holm-Sidak’s post-test in g and two-way ANOVA with and without repeated measures with Holm-Sidak’s post-test in d-e, respectively. **p<0.01, ***p<0.001, and ****p<0.0001.

Male C57BL/6J mice (Jackson Laboratories, Bar Harbor, ME) were 8–10 weeks old at the start of the study and received either a single subcutaneous (SQ) injection (acute dosing) or were injected SQ three days a week for four weeks (chronic dosing) with the TfRMAb-EPO at low (1 mg/kg), mid (3 or 6 mg/kg) or high (20 mg/kg) doses (n=4–5 per dose). Age-matched C57BL/6J littermate controls were treated with an equivalent volume of saline (SQ) using the same dosing regimen as described above (Fig. 1a).

For the acute dosing study, blood samples were collected at 3 hours and 6 hours after the single injection, and mice were euthanized 24 hours later. For the chronic dosing study, blood was collected weekly, 6 hours after TfRMAb-EPO injection, and mice received a terminal dose of TfRMAb-EPO or saline at 4 weeks after treatment initiation. Blood samples were collected at 3 hours and 6 hours after injection, and mice were euthanized 24 hours after the terminal injection. Plasma data at 3, 6, and 24 hours were used to calculate the area under the curve from 0–24 hours (AUC_0–24_) using the trapezoid method. At termination, blood was collected for hematocrit and metabolic panel under isoflurane anesthesia, mice were euthanized with a lethal dose of Euthasol (150 mg/kg, intraperitoneally (IP)) and underwent cardiac perfusion using ice-cold phosphate-buffered saline (PBS) for 5 min to clear the vasculature before organs were harvested (brain, kidney, liver, and spleen). Organs were snap-frozen in liquid nitrogen and used for ELISA and Western blot analysis.

A separate set of male C57BL/6J mice were treated chronically with the 20 mg/kg dose (n=5) or equivalent saline (n=5) for 4 weeks and then taken off treatment for 8 weeks. After 4 weeks of treatment and 8 weeks of wash-out (4 weeks on 8 weeks off), all mice received a final SQ dose of TfRMAb-EPO, blood samples were collected at 3, 6, and 24 hours, and mice were euthanized for tissue harvestation, as described above.

### APP_SAA_ KI Mice Study

Male APP_SAA_ KI mice (B6. Cg-Apptm1.1Dnli/J Strain: 034711, Jackson Laboratories, Bar Harbor, ME), which harbor the Swedish, Arctic, and Austrian (SAA) familial AD mutations [10], were 5.5 months of age before initiation of SQ injections three days a week for fourteen weeks, either with vehicle (n=5) or the TfRMAb-EPO fusion protein at a 1 mg/kg dose (n=6). Age-matched APP_WT_ littermate controls (n=4) (B6.Cg-Appem1Adiuj/J Strain: 033013, Jackson Laboratories, Bar Harbor, ME) without the SAA mutations, were treated with an equivalent volume of vehicle SQ using the same dosing regimen as described above (Fig. 1a). After fourteen weeks of treatment, open-field and Y-maze testing were performed, mice were anesthetized with a lethal dose of Euthasol (150 mg/kg, IP), and underwent cardiac perfusion using ice-cold PBS. The left hemi-brains were snap-frozen in liquid nitrogen and used for ELISA and Western blot analysis, and the right hemi-brains were fixed in 4% paraformaldehyde (PFA), cryoprotected with 10, 20, and 30% sucrose solutions, and used for immunostaining.

### Open-field test

The open-field test was done to assess the exploration and locomotor behavior of the APP_SAA_ and APP_WT_ mice according to the method described previously [12]. The test was performed using a square-shaped open-field box (90cm × 90 cm with 40 cm walls divided into four equal arenas (Panlab, Harvard Apparatus, Holliston, MA, USA). Briefly, a mouse was placed in each of the four arenas of the apparatus, followed by a video recording of mouse movements for 5 minutes. Mean speed and distance traveled by each mouse were evaluated using the SMART Video Multi Arena Tracking Software (Panlab, Harvard Apparatus, Holliston, MA, USA). All the animals were allowed to acclimatize to the experimental room for approximately 30 minutes before the experiment, and to avoid odor cues, the testing apparatus was wiped with 70% ethanol between tests.

### Y-maze test

The spatial reference memory of the APP_SAA_ KI or APP_WT_ mice treated with TfRMAb-EPO or vehicle was assessed using the Y-maze test according to the methods reported previously [12]. The Y-maze apparatus consists of three equal radial arms (30 cm length × 6 cm width with 15 cm height) placed at the ground level. The test was performed in two phases, i.e., the training and testing phases. In the training phase, after blocking the novel arm, the mouse was placed in the start arm and allowed to explore the start arm and familiarize itself for 8 minutes. After 30 minutes, the blocked novel arm was opened, and the mouse was allowed to explore all three arms for an additional 8 minutes. The discrimination index, which is the percentage of entries in the novel arm divided by the total number of entries in the novel and familiar arm combined, was quantified using the SMART Video Tracking Software (Panlab, Harvard Apparatus, Holliston, MA, USA).

### Nest building

The nest building is a spontaneous behavior in mice, and it is indicative of the level of self-care and motivation, and is used widely to assess the overall well-being [13] and apathy-like behavior [14] in mice. All the animals were housed in single cages placed with pre-weighed Nestlets (Newco Specialty, CA, USA) weighing ~2.5 g. The following day, nesting scores were recorded, as described previously, by two observers blinded to the experimental groups [9].

### Brain sectioning

The 4% PFA-fixed and cryoprotected frozen right hemi-brains were mounted in the Leica CM1850 cryostat (Leica, Wetzlar, Germany), maintained at −20°C using Tissue-Tek OCT compound (Fisher Scientific, MA, USA), followed by either coronal or sagittal sectioning of the brains at 20μm thickness. The brain sections collected in PBS solution containing 0.01% sodium azide were stored at 4°C until immunostaining. For each mouse, four sagittal sections 600 μm apart were used for immunostaining [12].

### 6E10 immunostaining and quantification

Aβ deposits in the brain sections were stained with Alexa Fluor 488-labelled monoclonal antibody (BioLegend, CA, USA) that is directed against human Aβ (1–16) as described previously [12]. In brief, free-floating sagittal brain sections washed in PBS were subjected to antigen retrieval with 70% formic acid, followed by water wash and blocking with 0.5% bovine serum albumin (BSA) in PBS containing 0.3% Triton X-100 for 60 min at room temperature. Sections were stained with the 6E10 primary antibody solution (1:1000) overnight at 4°C. The brain sections washed in PBS, followed by water wash, were mounted onto glass slides, air dried, and coverslipped using Vectamount aqueous mounting media (Vector Laboratories, CA, USA), sealed with nail polish, and stored at 4 °C until further imaging. Full brain section images were obtained using the BZ-X710 Keyence fluorescence microscope (Keyence, IL, USA). Brain section images were analyzed and quantified for the total Aβ-positive counts, Aβ-positive area, and Aβ-stain size using the NIH ImageJ software (Bethesda, MD, USA) by two readers blinded to the treatment groups.

### Iba1 and TREM2 dual immunostaining and quantification

Ionized calcium-binding adaptor molecule-1 (Iba1) is a microglial marker, and triggering receptor expressed on myeloid cells-2 (TREM2) is an immune receptor found in microglia [15]. Four free-floating brain sections of 20μm thickness per mouse brain were used for dual staining of Iba1 and TREM2. For the antigen retrieval process, the brain sections were washed with PBS and incubated in sodium citrate buffer (10 mM sodium citrate acid, 0.05% Tween 20, pH 6.0) for 15 min at 90°C. Brain sections were washed with deionized water, followed by blocking with a mixture of 0.5% BSA and 0.3% Triton X-100 in PBS for 1 hour. Post blocking, the brain sections were incubated overnight at 4°C with gentle shaking in a cocktail mixture of primary antibodies, i.e., anti-Iba1 rabbit antibody (1:1000 #019–19741, Fujifilm Wako) and anti-TREM2 sheep antibody (1:250, #AF1729, R&D System, Minneapolis, USA) prepared in 0.5% BSA with 0.3% Triton X-100 in PBS. On the next day, after PBS wash, the brain sections were incubated for 2 hours at room temperature with gentle shaking in a cocktail mixture of secondary antibodies, i.e., antirabbit Alexa Fluor 488 for Iba1 (1:500, #40641, BioLegend, CA, USA) or anti-sheep Alexa Fluor 647 for TREM2 (1:500, #A21448, Thermo-Fisher Scientific, CA, USA) prepared in 0.5% BSA with 0.3% Triton X-100 in PBS. Brain sections were washed with distilled water and mounted on the slides, followed by cover slipping using Vectamount aqueous mounting media (Vector Laboratories, CA, USA) and sealing with nail polish. The slides were stored in the dark at 4°C until imaging. The Iba1 and TREM2 co-stained brain sections were imaged using a Nikon Ti-E Confocal Microscope (Nikon Instruments Inc, NC, USA) with NIS Element software. The laser and detector settings were standardized and kept constant throughout the imaging process. The images were captured under a 40X oil immersion lens at 1024×1024 pixels. Two distinct regions in each of the cortex and hippocampus, and one distinct region in the amygdala, from four mouse brain sections per mouse, were imaged and analyzed using NIH ImageJ using a threshold setting to calculate tissue area positive for Iba1 and TREM2.

### Preparation of brain homogenates for APP_SAA_ KI and APP_WT_ mice

The frozen left-brain hemispheres were pulverized under dry ice, and a portion of the pulverized tissues was homogenized with tris-buffered saline (TBS, 1:10 w/v) containing 50 mM Tris-HCl, pH 7.6, 150 mM NaCl, 5 mM EDTA, 2 mM 1,10-phenanthroline, and Roche complete EDTA-free Mini protease inhibitor. The brain homogenates were centrifuged at 100,000 g for 1 h at 4°C, followed by collecting the supernatants (TBS-soluble fractions) into multiple aliquots to avoid multiple freeze-thaws and were stored at −80°C until further analysis. The remaining pellets were resuspended in 10 volumes of homogenizing buffer (5 M guanidine HCl (Gu-HCl), 0.05 M Tris, pH 8.0) and homogenized. The brain homogenates were then allowed to shake at room temperature for 2 hours, followed by centrifugation at 20,800 g for 15 min at room temperature, and collecting the supernatants (Gu-HCl-soluble fractions) into multiple aliquots to avoid repeated freeze-thaw. Samples were stored at −80°C until further analysis. Further, a portion of the pulverized brain samples were homogenized at a ratio of 1:15 (w/v) of radioimmunoprecipitation assay buffer (RIPA) containing Roche complete EDTA-free Mini protease inhibitor, followed by constant shaking on a rotor for 1 hour at 4°C and centrifuging the mixture at 12,000 g for 20 min at 4°C. Later the supernatants (RIPA fractions) were collected in multiple aliquots and stored at −80°C until further analysis. The protein content of the brain (TBS- and Gu-HCl-soluble and RIPA) fractions was determined using the bicinchoninic acid (BCA) method (Pierce Chemical Co., Rockford, IL, USA).

### Quantification of organ and plasma TfRMAb-EPO concentrations

Plasma was separated from whole blood samples, and frozen organs (spleen, kidney, liver, and brain) were pulverized into a fine powder in a cold room and homogenized in Tissue Protein Extraction Reagent (TPER) buffer (ThermoScientific, Rockford, IL, US) containing EDTA-free protease inhibitors (Roche Diagnostics, Mannheim, BW Germany) using 5 μL of T-PER per mg of tissue for the kidney, liver, and spleen, and 2 μL of T-PER per mg of brain. Homogenates were rotated for 1 hour, followed by centrifugation at 14,000 g for 20 minutes at 4°C. Supernatants were aliquoted into labeled Eppendorf vials and stored at −80°C for further analysis.

96-well Nunc Maxisorp plates (Fisher Scientific, Waltham, MA, US) were coated with 200 ng/well EPOR/Fc (R&D System, Minneapolis, USA) working solution and incubated overnight. The next day, the solution was aspirated, and wells were washed three times with 150μL of Tris-buffered saline with 0.05% Tween-20 (TBST), followed by blocking with 150μL of Tris-buffered saline containing 0.1% bovine serum albumin (TBSB) for 30 minutes at room temperature. After aspirating the TBSB solution, the standards or the test samples (100 μL per well) were added to their respective wells and incubated for 2 hours at room temperature. After aspiration and washing with TBST, 100 μL of goat anti-mouse light chain (kappa) antibody conjugated to alkaline phosphatase (GAM-AP) (Bethyl Laboratories, MA, USA) working solution (100 ng/well) was added and incubated for 45 minutes at room temperature. Wells were washed again, followed by the addition of 100 μL p-Nitrophenyl Phosphate (PNPP, Sigma-Aldrich, St. Louis, MO, USA) working solution. After incubating for 5–15 minutes in the dark (development time adjusted as needed), the reaction was stopped with 100 μL of 1.2 M NaOH. Absorbance was measured at 405 nm using a plate reader [16, 17].

To determine the concentration of TfRMAb-EPO in brain homogenates, a Meso Scale Discovery (MSD) electrochemiluminescence assay was performed using the MSD GOLD^™^ 96-well Small Spot Streptavidin SECTOR 96-well plate (Meso Scale Diagnostics, Rockville, MD, US). The plate was blocked with 150 μL per well of TBS with 3% BSA (Roche Diagnostics GmbH, Mannheim, BW, Germany) and incubated at room temperature for 1 hour at 900 rpm shaking. After blocking, wells were aspirated, and 30 μL of biotinylated EPOR (BPS Bioscience, San Diego, CA, US) at 0.25 μg/mL in TBS with 1% BSA was added to each well. The plate was incubated for 1 hour at room temperature with shaking (900 rpm), followed by three washes with 150 μL per well of TBST. Brain homogenates and standards (25 μL) were added per well, and the plate was incubated for 1 hour at room temperature with shaking (900 rpm). After incubation, wells were washed three times with 1×TBST. For detection, 25 μL of 1 μg/mL goat anti-mouse sulfo-tag (MSD, Rockville, MD, US) in 1% BSA in TBS at 25 ng/well was added and incubated for 1 hour at room temperature with shaking (900 rpm). After washing three times with 1×TBST, 150 μL of Gold Read Buffer B (MSD, Rockville, MD, US) was added. The plate was read using an MSD MESO Quickplex SQ 96-well plate reader (MSD, Rockville, MD, US), and the data was collected and analyzed.

### Erythropoietin Receptor (EPOR) ELISA

To determine the changes in organ levels of EPOR in mice treated with the TfRMAb-EPO fusion protein, liver, spleen, and kidney homogenates were prepared in TPER buffer (Thermo Scientific, Rockford, IL, US) supplemented with EDTA-free protease inhibitors (Roche Diagnostics, Mannheim, BW, Germany) as described above. EPOR levels in the tissue supernatant were quantified using an ELISA method following the manufacturer’s protocol (NBP2–67948, Novus Biologicals, Centennial, CO, US). Absorbance was measured at 450 nm, and EPOR concentrations were determined based on the standard curve.

### Quantification of anti-drug antibodies (ADA)

96-well Nunc Maxisorp plates (Fisher Scientific, Waltham, MA, US) were coated with 100 μL of TfRMAb-EPO (capture agent) working solution (300 ng/well) and incubated overnight at 4°C. The next day, wells were washed three times with PBS containing 1% BSA (PBSB) (150 μL) and blocked with 150 μL PBSB for 30 minutes at room temperature. After aspiration, 100 μL of 50-fold diluted plasma samples were added and incubated for 1 hour at 37°C. Wells were washed, then incubated with 100 μL of biotinylated TfRMAb-EPO (50 ng/well), which was biotinylated using a biotinylation kit (Thermo-Fisher Scientific, CA, USA), for 1 hour at 37°C, followed by another PBSB wash. Streptavidin-alkaline phosphatase (SA-AP) working solution (Thermo-Fisher Scientific, CA, USA) was added (0.5 μg/well) and incubated for 30 minutes at room temperature. After washing, 100 μL of PNPP (Sigma-Aldrich, St. Louis, MO, USA) working solution was added and incubated for 5–15 minutes in the dark. The reaction was stopped with 100 μL of 1.2 M NaOH, and absorbance (optical density, OD) was measured at 405 nm using a plate reader. ADA was reported as the signal-to-noise ratio (SNR), which is the OD in the TfRMAb-EPO-treated plasma divided by the OD in the saline-treated plasma [18].

### Quantification of TfR by ELISA

TfR levels in plasma and homogenized tissues were quantified using a mouse TfR ELISA kit (ab243674, Abcam, Cambridge, MA, USA). Supernatants were diluted 25- to 250-fold, and the assay was run as per the vendor’s instructions.

### Quantification of aggregated human-Aβ by ELISA

The aggregated human Aβ (hAβ) was measured in the TBS-soluble brain fractions (diluted 2-fold) and Gu-HCl-soluble brain fractions (diluted 10-fold) using the commercially available ELISA kit (#KHB3491, Thermo-Fisher Scientific, CA, USA) as per the vendor’s instructions.

### Quantification of mouse brain-derived neurotrophic factor (BDNF) by ELISA

The mouse BDNF levels were measured in the TBS-soluble brain fractions and plasma samples of APP_SAA_ KI or APP_WT_ mice treated with TfRMAb-EPO or vehicle using the commercially available ELISA kit (#EEL088, Thermo-Fisher Scientific, CA, USA) as per vendor’s instructions.

### Western blot analysis

The expression of zonula occludens-1 (ZO-1), postsynaptic density protein-95 (PSD-95), beta-site amyloid precursor protein cleaving enzyme 1 (BACE1), and presenilin 1 (PSN-1) in the brain fractions of the vehicle control- or TfRMAb-EPO-treated APP_SAA_ KI or APP_WT_ mice was determined by Western blotting. Briefly, the TBS-soluble and RIPA-soluble brain fractions (~25 μg protein/lane) were processed in the 4x-lamelli buffer and separated by SDS-polyacrylamide gel electrophoresis using precast 4–20% MP TGX gels (Bio-Rad, Hercules, CA, USA) at a constant voltage of 100 V for 60 min. Proteins from the gel were transferred onto a polyvinylidene fluoride (PVDF) membrane (0.45 μm) using the Tank Transfer System (Bio-Rad, Hercules, CA, USA), followed by blocking of the PVDF membrane by incubating in 3% milk for one hour at room temperature. After blocking, the blots were incubated overnight at 4°C with their respective primary antibodies diluted in 3% milk in 1x TBS: ZO-1 (1:1000, #40–2200, Bio-Legend, CA, USA), BACE1 (1:1000, #PA1–757, Invitrogen, CA, USA), PSD-95 (1:1000, #sc-32290, Santa Cruz, CA, USA), PSN1 (1:1000, #5643, Cell Signaling, MA, USA), or β-actin (loading control, 1:1000, #sc-47778, Santa Cruz, CA, USA). After washing, the blots were incubated with their respective HRP-conjugated IgG anti-rabbit (1:1000, #7074, Cell Signaling, MA, USA), or HRP-conjugated anti-mouse IgG kappa (1:1000, #sc-516102, Santa Cruz, CA, USA), secondary antibodies for one hour at room temperature, followed by washing before visualization of bands with the Bio-Rad Chemi Doc Imager system (Bio-Rad, Hercules, CA, USA). NIH ImageJ (version 1.53e, MD, USA) was used to quantify the intensity of the Western blot bands, protein expression was normalized to the β-actin protein expression, and the data were represented as % of the control-treated group [19].

### Statistical analysis

The data were expressed as mean ± SEM of n =4–6 per group and analyzed using GraphPad Prism (v10.04, La Jolla, CA, USA). Sample size estimation was performed using G*Power to detect a predicted effect size of 40% with a standard deviation of 15–20%, 80% power, and a significance level of 5%, for PK studies, based on our previous work [11], which resulted in 4–6 mice per group. For the AD mouse study, with a predicted effect size of 50% (since we wanted to see a greater effect than our previous work with TfRMAb-EPO with the 3 mg/kg dose [8, 9], which results in a lower brain uptake than the 1 mg/kg dose that was used herein (see results section)), a standard deviation of 20–25%, 80% power, and a significance level of 5%, the sample size was between 4–6 mice per group. For a continuous numerical dependent variable, one-way analysis of variance (ANOVA) for one independent variable or two-way ANOVA for two independent variables, followed by Holm’s Sidak post-hoc test, was used. Outliers were excluded using Grubb’s outlier test. For unequal variances, the Welch’s ANOVA was used. Correlation was analyzed using the Pearson correlation coefficient *r*. A two-tailed p-value ≤ 0.05 was considered statistically significant.

## RESULTS

### Plasma pharmacokinetics of TfRMAb-EPO

Plasma concentrations of TfRMAb-EPO after acute and chronic dosing are presented in Fig. 1b–c. Following acute dosing, plasma concentrations increased in a dose dependent manner, and all four doses (1, 3, 6, and 20 mg/kg) demonstrated a maximum plasma concentration (C_max_) at 6 hours post TfRMAb-EPO administration (Fig. 1b). In contrast, chronic dosing at 3, 6, and 20 mg/kg showed markedly reduced plasma TfRMAb-EPO concentrations and AUC_(0–24)_ compared to their acute dosing counterparts (Fig. 1b, c, and e). Plasma concentrations of TfRMAb-EPO declined within a week of repeated dosing at 3, 6, and 20 mg/kg doses, but the decline in plasma exposure was reduced with an increase in injected dose (Fig. 1d). Notably, mice treated with 1 mg/kg of TfRMAb-EPO exhibited no significant reduction in plasma concentration or AUC_(0–24)_ after four weeks of chronic dosing (Fig. 1d–e). Plasma concentrations and AUC_0–24_ of TfRMAb-EPO continued to be significantly lower (p<0.001) in the mice chronically dosed with 20 mg/kg TfRMAb-EPO for four weeks, followed by an eight-week washout period and a final 20 mg/kg TfRMAb-EPO dose, compared to mice that received saline for four weeks, followed by an eight-week washout period and a final 20 mg/kg TfRMAb-EPO dose (Fig. 1f–g). However, mice subjected to the washout showed significantly higher plasma AUC_0–24_ (p<0.01) of TfRMAb-EPO than mice that were dosed chronically for four weeks without a washout period, showing some recovery in plasma exposure following an extended drug-free period (Fig. 1f–g).

### Organ distribution of TfRMAb-EPO

**Figure 2:**
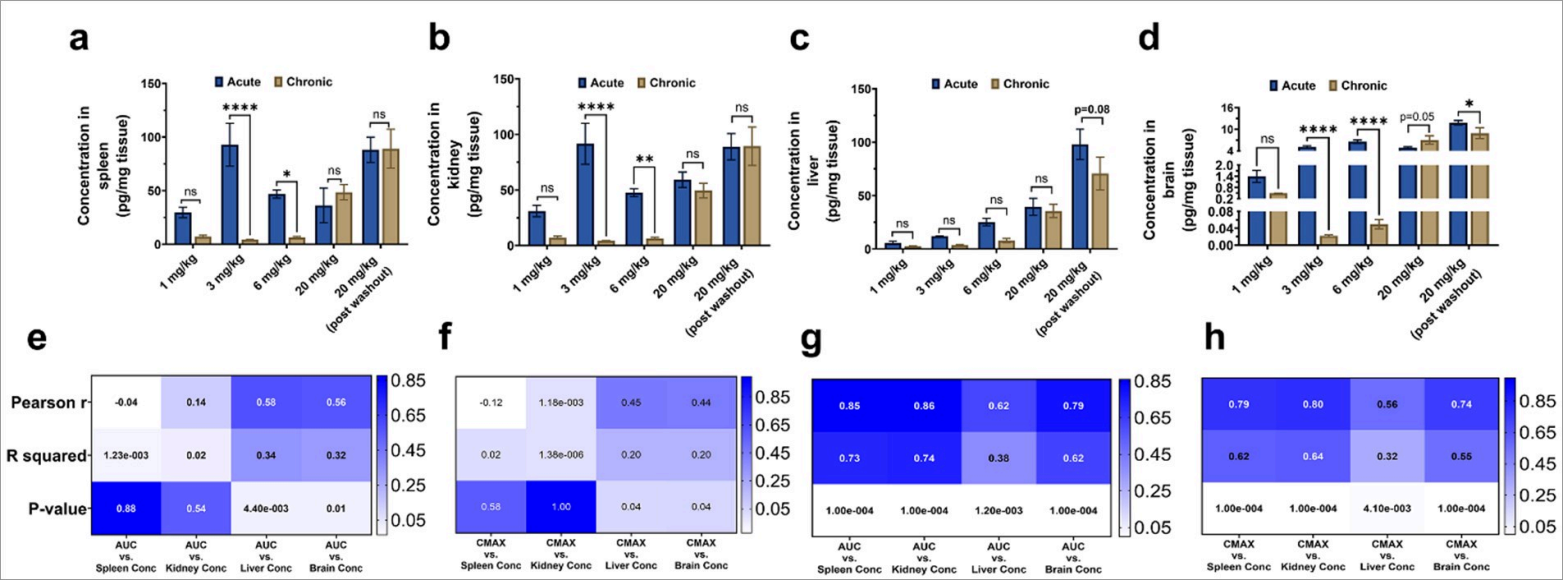
Biodistribution of TfRMAb following acute and chronic dosing. Organ concentrations of TfRMAb-EPO in both acutely and chronically dosed mice in the spleen (a), kidney (b), liver (c), and brain (d). Correlational analysis show a moderate correlation between liver and brain TfRMAb-EPO concentrations and both plasma area under the curve (AUC_0–24_) (e) and plasma C_max_ following acute dosing (f). For chronic dosing, a moderate correlation was observed between liver concentrations of TfRMAb-EPO and both plasma AUC_0–24_ (g) and plasma C_max_ (h). Both plasma AUC_0–24_ (g) and plasma C_max_ (h) were strongly correlated with spleen, kidney, and brain TfRMAb-EPO concentrations (*r* = 0.74–0.86). Data are represented as mean ± SEM of n = 4–6 per group and were analyzed using the two-way ANOVA with Holm-Sidak’s post-test in a-d and Pearson correlation in e-h, respectively. *p<0.05, **p<0.01, and ****p<0.0001. ns: non-significant.

The organ concentrations of TfRMAb-EPO following acute and chronic dosing are shown in Fig. 2. Acute dosing resulted in significantly higher TfRMAb-EPO levels in spleen, kidney, and brain compared to chronic dosing for 3 and 6 mg/kg doses, with a similar trend (without statistical significance) with the 1 mg/kg dose (Fig. 2a–d). A similar trend was observed in the liver, where the TfRMAb-EPO concentrations were higher after acute dosing compared to chronic dosing, but these values did not reach statistical significance. This trend was different at the 20 mg/kg dose, where the TfRMAb-EPO concentrations were comparable in the spleen, liver, kidney, and brain following acute and chronic dosing, possibly due to TfR saturation associated with the high TfRMAb-EPO doses (Fig. 2a–d). Following acute dosing, TfRMAb-EPO concentrations in the liver (Pearson *r* = 0.58, p<0.01) and brain (Pearson *r* = 0.56, p<0.01) were positively correlated with plasma exposure (C_max_ and AUC_0–24_), whereas the spleen and kidney TfRMAb-EPO concentrations were not correlated with TfRMAb-EPO plasma exposure (Fig. 2e–f). Following four weeks of chronic dosing, a significant moderate positive correlation was observed between liver TfRMAb-EPO concentrations and plasma AUC_0–24_ (p<0.01, Pearson *r* = 0.62, Fig. 2g) and C_max_ (p<0.01, Pearson *r* = 0.56, Fig.2h), whereas TfRMAb-EPO concentrations in spleen, kidney, and brain demonstrated a strong significant positive correlation (p<0.0001, Pearson *r* = 0.74–0.86) with plasma AUC_0–24_ (Fig. 2g) and C_max_ (Fig.2h).

### TfR levels in plasma and organs

**Figure 3:**
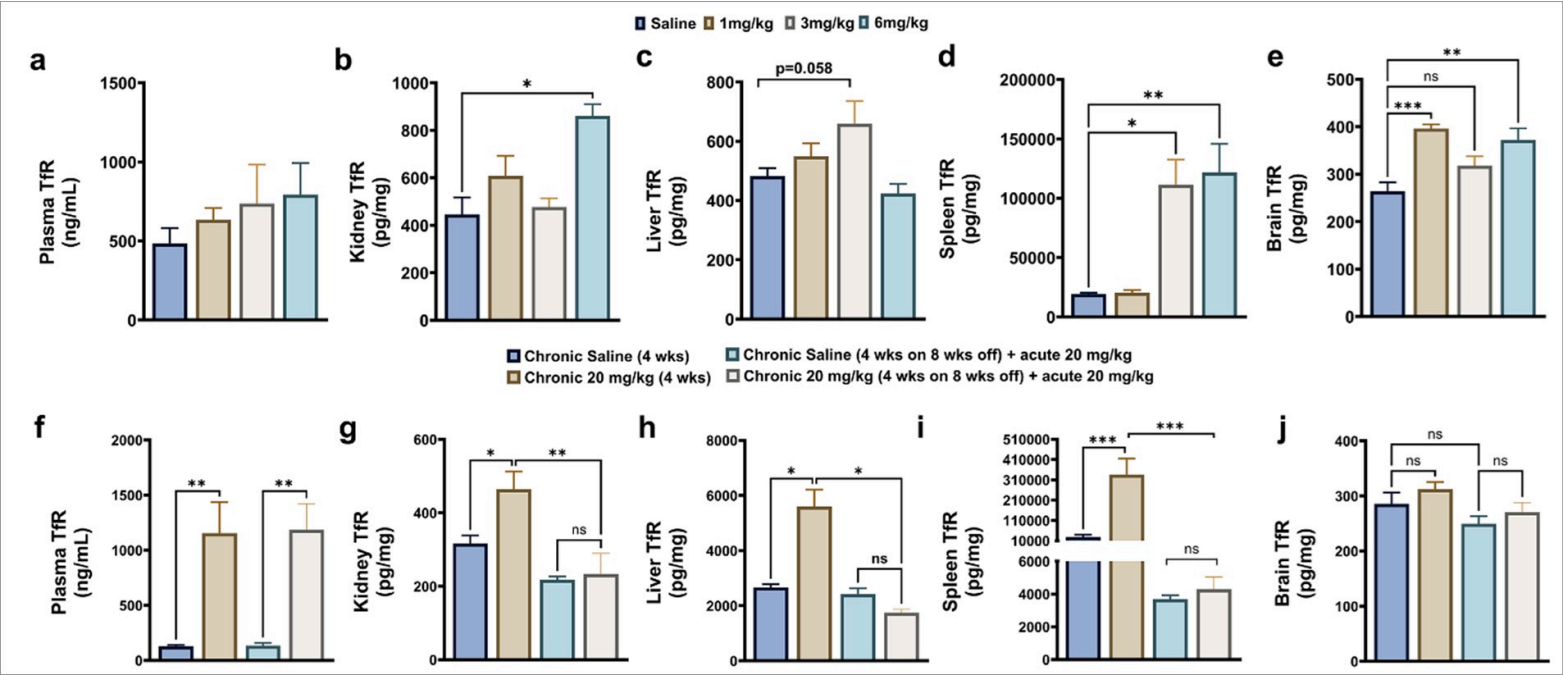
Alterations in transferrin receptor (TfR) concentrations following chronic dosing. TfR levels in plasma (a), kidney (b), liver (c), spleen (d), and brain (e) following 1, 3, and 6 mg/kg chronic doses of TfRMAb-EPO compared with saline-treated mice. Changes in TfR levels in the plasma (f), kidney (g), liver (h), spleen (i), and brain (j) following a 20 mg/kg chronic dose for four weeks and following a 20 mg/kg chronic dose for four weeks with an eight-week washout followed by a final 20 mg/kg dose, compared to saline-controls. Data are represented as mean ± SEM of n = 4–6 per group and were analyzed using the two-way ANOVA with Holm-Sidak’s post-test. *p<0.05, **p<0.01, and ***p<0.001. ns: non-significant.

TfR levels in plasma and organs following chronic TfRMAb-EPO dosing are shown in Fig. 3. No significant changes were detected in plasma, kidney, and liver TfR levels with the 1, 3, and 6 mg/kg TfRMAb-EPO doses in comparison to the saline control group (Fig. 3a–c), except for the kidney TfR levels which were significantly elevated with chronic 6 mg/kg dosing (p<0.05, Fig. 3b) with a similar trend in the liver TfR with the 3 mg/kg chronic dose (p=0.058, Fig. 3c). Spleen TfR levels remained unchanged with the 1 mg/kg dose but were 6-fold higher with chronic 3 mg/kg (p<0.05) and 6 mg/kg (p<0.01) dose (Fig. 3d). TfR levels in the brain were significantly elevated with chronic 1 mg/kg (p<0.001) and 6 mg/kg (p<0.01) doses, with a similar trend with the 3 mg/kg dose (Fig. 3e).

At the high TfRMAb-EPO chronic dose of 20 mg/kg, TfR levels were significantly elevated in the plasma (p<0.01), kidney (p<0.05), liver (p<0.05), and spleen (p<0.001), but not in the brain (Fig. 3f–j). Interestingly, these elevated TfR levels returned to normal values with an eight-week washout period in the kidney, liver, and spleen, but not in the plasma (Fig. 3f–i).

### ADA response

**Figure 4:**
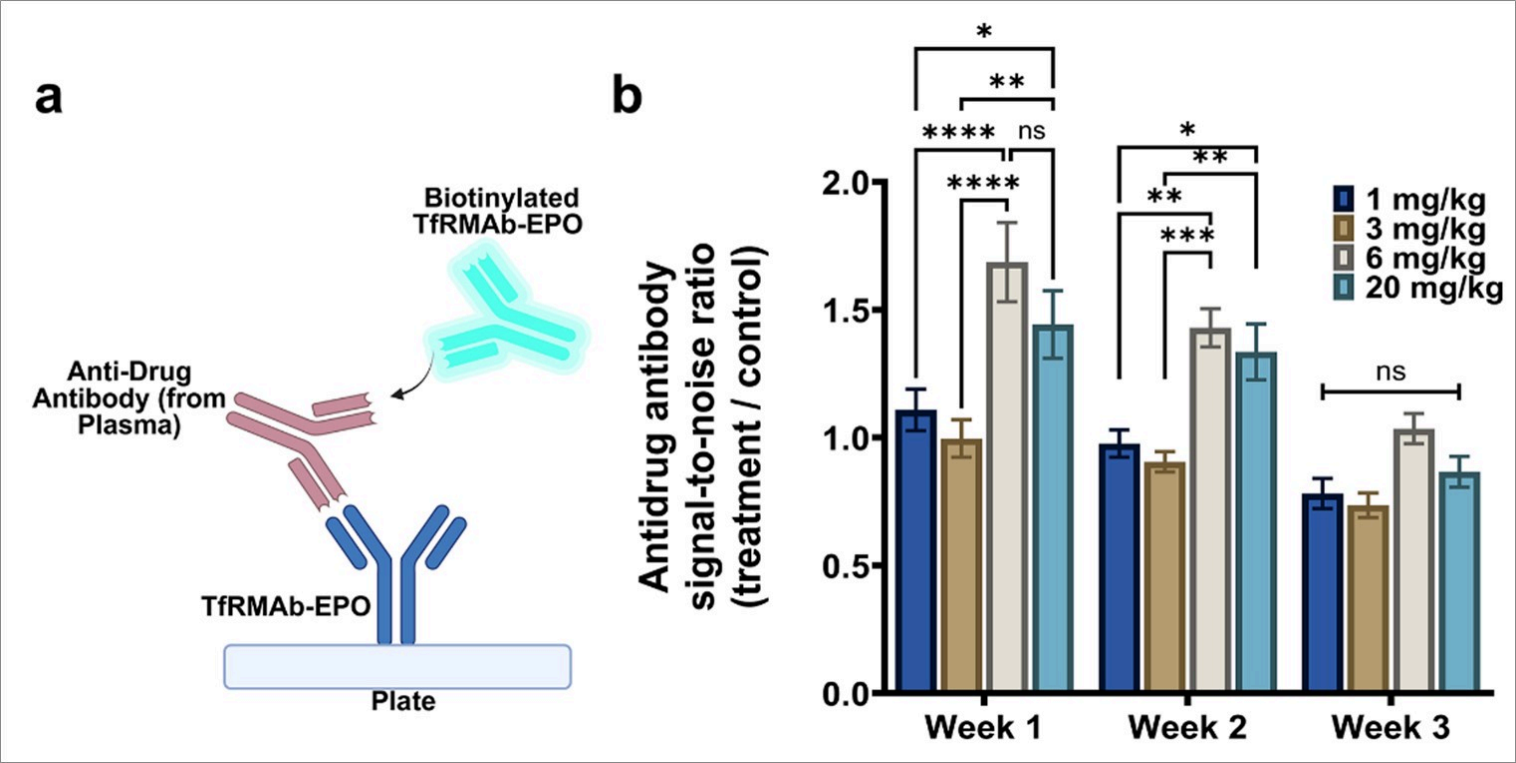
Anti-drug antibody formation following chronic dosing. Weekly anti-drug antibody (ADA) response against TfRMAb-EPO was evaluated following chronic dosing using a sandwich ELISA, with TfRMAb-EPO serving as the capture agent and biotinylated TfRMAb-EPO serving as the detector agent (a). ADA was reported as the signal-to-noise ratio (SNR), which is the OD in the TfRMAb-EPO-treated plasma divided by the OD in the saline-treated plasma (b). Data are represented as mean ± SEM of n = 4–6 per group and were analyzed using the two-way repeated measures ANOVA with Holm-Sidak’s post-test. *p<0.05, **p<0.01, ***p<0.001, and ****p<0.0001. ns: non-significant.

ADA response to chronic TfRMAb-EPO dosing was evaluated using a sandwich ELISA (Fig. 4a). After one (three injections) and two (six injections) weeks of repeated dosing, SNR values were significantly elevated with the 6 mg/kg and 20 mg/kg doses compared to the 1 mg/kg and 3 mg/kg doses (Fig. 4b). Three weeks after repeated dosing, the SNR values did not differ between any doses (Fig. 4b).

### Hematocrit levels and spleen weights following acute and chronic dosing regimens

**Figure 5:**
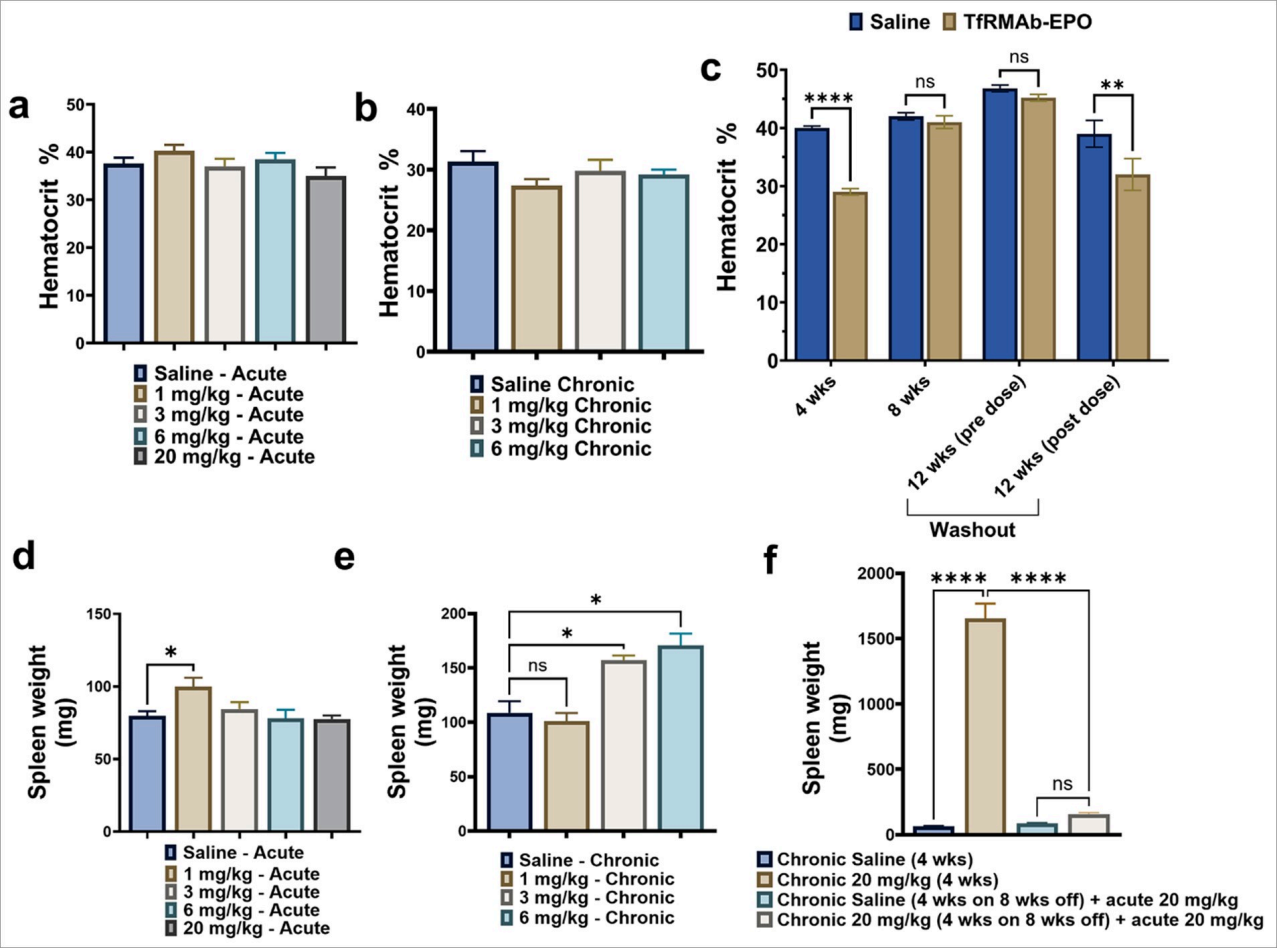
Hematocrit and spleen weights. Hematocrit (%) measurements in mice following acute (a) or chronic (b) administration of TfRMAb-EPO at 1, 3, and 6 mg/kg show no significant changes compared to saline controls. A significant reduction in hematocrit following 4 weeks of repeated 20 mg/kg TfRMAb-EPO dosing (c), followed by recovery of hematocrit to normal levels during the eight-week washout period compared to saline controls (c). A single final terminal dose of 20 mg/kg TfRMAb-EPO, following four weeks of chronic dosing and eight weeks of washout, significantly reduced hematocrit (c) compared to saline-treated mice that received one final 20 mg/kg dose of TfRMAb-EPO. Spleen weights following acute dosing (d), chronic dosing (e), and chronic dosing with an eight-week washout and a final TfRMAb-EPO dose (f). Data are represented as mean ± SEM of n = 4–6 per group and were analyzed using the one-way ANOVA with Holm-Sidak’s post-test in a, b, d, e, and f, and two-way repeated measures ANOVA with Holm-Sidak’s post-test in c, respectively. *p<0.05, **p<0.01, and ****p<0.0001. ns: non-significant.

Acute and chronic administration of TfRMAb-EPO at 1, 3, and 6 had no significant effect on hematocrit levels (Fig. 5a–b). A single acute high 20 mg/kg dose of TfRMAb-EPO did not alter hematocrit; however, four-week chronic dosing at this high dose significantly decreased hematocrit (10% reduction, p<0.0001) compared to saline control, which was reversed during the eight-week washout period (Fig. 5c). Interestingly, a single final 20 mg/kg dose of TfRMAb-EPO to mice that were dosed with TfRMAb-EPO for four weeks followed by an eight-week washout was sufficient to significantly reduce hematocrit (7% reduction, p<0.01) compared to mice that were dosed with saline for four weeks followed by an eight week washout and a final TfRMAb-EPO dose (Fig. 5c).

Spleen weights following acute TfRMAb-EPO treatment significantly increased in the 1 mg/kg group (25% increase, p<0.01), while other acute doses showed no change (Fig. 5d). Chronic dosing at 3 mg/kg (p<0.05), 6 mg/kg (p<0.05), and 20 mg/kg (p<0.0001) resulted in significant increase in spleen weight compared to controls (Fig. 5e–f), which returned to normal after the eight-week washout (Fig. 5f). Kidney weights remained unchanged across all acute and chronic groups (Supplemental Figure 1), suggesting no renal enlargement or overt renal changes.

### Effect of TfRMAb-EPO on locomotion and spatial reference memory of APP_SAA_ KI mice

**Figure 6:**
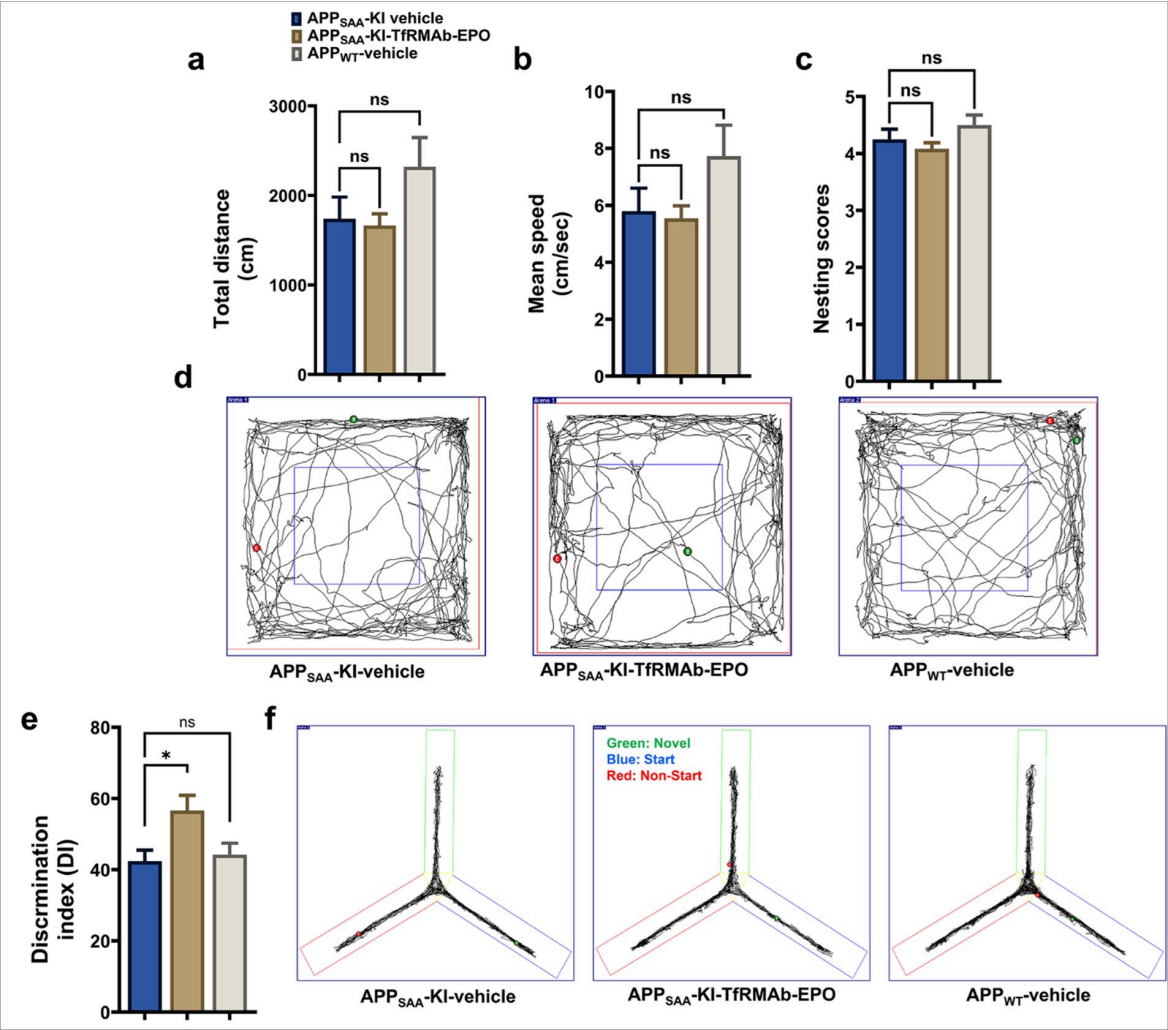
Effect of chronic TfRMAb-EPO treatment on exploratory behavior and spatial reference memory of APP_SAA_ KI mice. No significant change in the total distance travelled (a), and mean speed (b) between the vehicle-treated and TfRMAb-EPO-treated APP,_SAA_-KI mice. TfRMAb-EPO treatment showed no significant change in nesting behaviors in APP_SAA_ KI mice compared to the vehicle-treated APP_SAA_ KI mice (c). Representative open-field trajectory maps are shown in d. A significant increase in the discrimination index for the novel arm for the APP_SAA_ KI mice treated with TfRMAb-EPO compared to their respective control groups (e). Representative Y-maze trajectory maps are shown in f. Data are represented as mean ± SEM of n = 4–6 per group and were analyzed using the one-way ANOVA with Holm-Sidak’s post-test. *p<0.05. ns: non-significant.

The effect of TfRMAb-EPO on the locomotion and overall well-being of APP_SAA_ KI mice was evaluated using the open-field test and nesting behavior, respectively. APP_SAA_ KI mice generally showed reduced locomotor indices, which did not reach statistical significance, with no significant differences in nesting behavior, compared to the APP_WT_ mice (Fig. 6a–c). TfRMAb-EPO treatment did not show any significant change in the total distance and mean speed of APP_SAA_ KI mice (Fig. 6a, b) compared to the vehicle-treated APP_SAA_ KI or APP_WT_ mice. Similarly, no significant change in the nesting scores was observed between the TfRMAb-EPO-treated and vehicle-treated APP_SAA_ KI or APP_WT_ mice (Fig. 6c). Representative trajectory maps tracking the mouse movements in the open-field arena are shown in Fig. 6d. The APP_SAA_ KI mice treated with TfRMAb-EPO showed a significantly higher discrimination index for the novel arm compared with the familiar arm (p<0.05, Fig.6e) in the Y-maze compared to that of vehicle-treated APP_SAA_ KI or APP_WT_ mice. Representative trajectory maps showing the mouse movement in the Y-maze arena are shown in Fig. 6f.

### Effect of chronic TfRMAb-EPO treatment on Aβ load in the brains of APP_SAA_ KI mice

**Figure 7:**
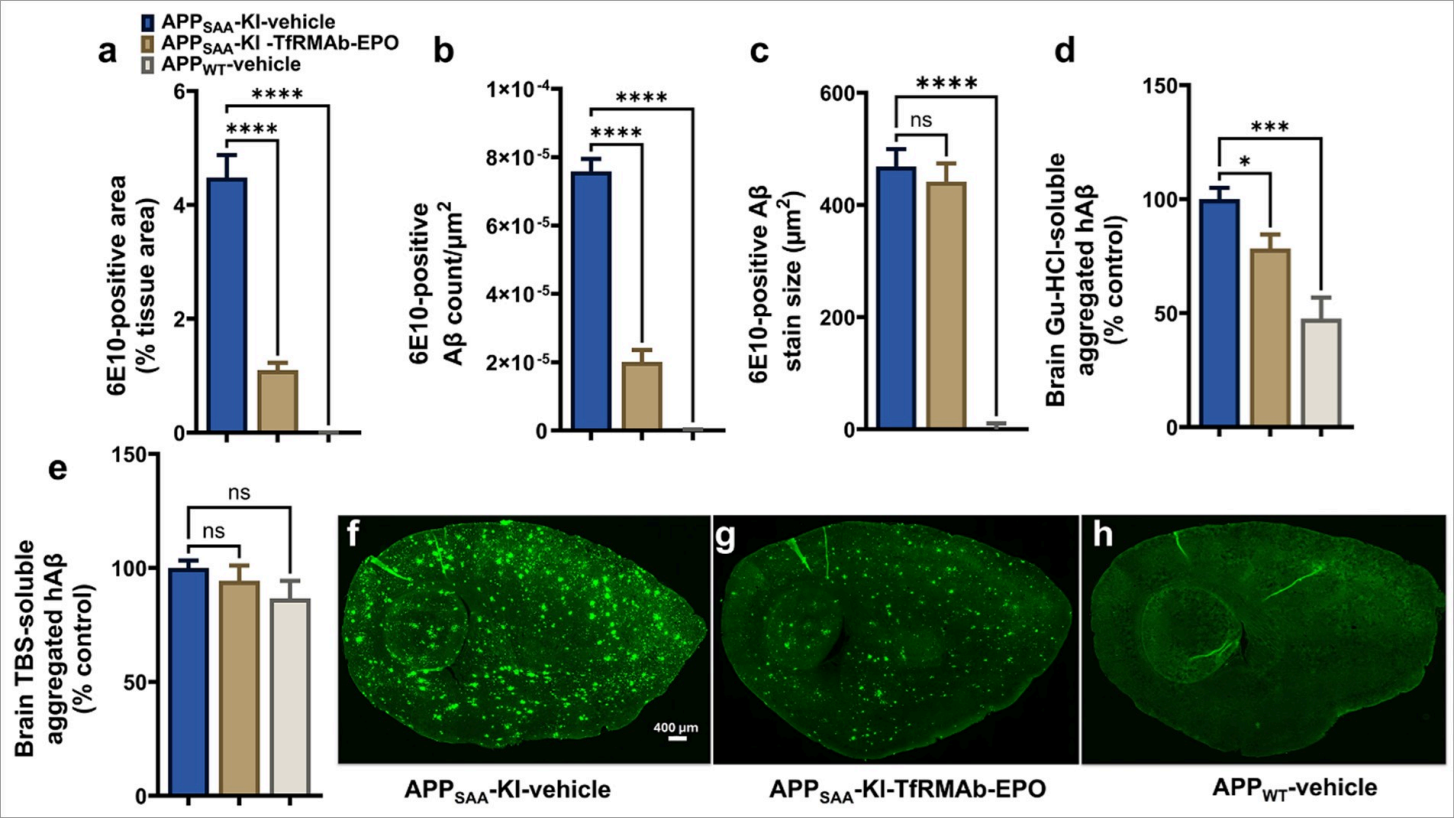
Effect of chronic TfRMAb-EPO treatment on the brain Aβ load in APP_SAA_ KI mice. There was a significant decrease in the 6E10-positive area (a) and 6E10-positive Aβ-count (b), with no change in the 6E10-positive Aβ-stain size (c), in the TfRMAb-EPO-treated APP_SAA_ KI mice compared to vehicle-treated APP_SAA_ KI mice. ELISA data showed a significant decrease in the Gu-HCl-soluble aggregated human Aβ (hAβ) levels (d) with no significant change in the soluble aggregated hAβ levels (e) in the TfRMAb-EPO-treated APP_SAA_ KI mice compared to vehicle-treated APP_SAA_ KI mice. Representative sagittal brain section images of 6E10-positive Aβ stains of vehicle-treated APP_SAA_ KI mice (f), TfRMAb-EPO-treated APP_SAA_ KI mice (g), and vehicle-treated APP_WT_ mice, respectively (h). Data are represented as mean ± SEM of n = 4–6 per group and were analyzed using the one-way ANOVA with Holm-Sidak’s post-test. *p<0.05, ***p<0.001, ****p<0.001. Scale bar = 400 μm. ns: non-significant.

The effect of chronic TfRMAb-EPO treatment on Aβ pathology in the APP_SAA_ KI mice was assessed using 6E10-immunostaining and ELISA. As expected, no Aβ deposits were observed in the APP_WT_ mice [10]. A profound significant decrease in the 6E10-positive area (77% reduction, p<0.0001, Fig. 7a) and 6E10 count (73% reduction, p<0.0001, Fig. 7b), with no change in the average 6E10 stain size (Fig. 7c), was observed with TfRMAb-EPO treatment compared with vehicle-treated APP_SAA_ KI mice. Consistent with these observations, there was a significant decrease in the insoluble aggregated human Aβ levels (p<0.05, Fig. 7d) between the TfRMAb-EPO-treated and vehicle-treated APP_SAA_ KI mice brains. No significant change was observed in the soluble aggregated human Aβ levels in the brains of mice treated with TfRMAb-EPO compared to vehicle-treated APP_SAA_ KI mice (Fig. 7e). The representative 6E10stained brain sections are shown respectively in Fig. 7f–h.

### Effect of TfRMAb-EPO on BDNF, and proteins involved in synaptic health, Aβ production, and BBB tightjunction in APP_SAA_ KI mice

**Figure 8:**
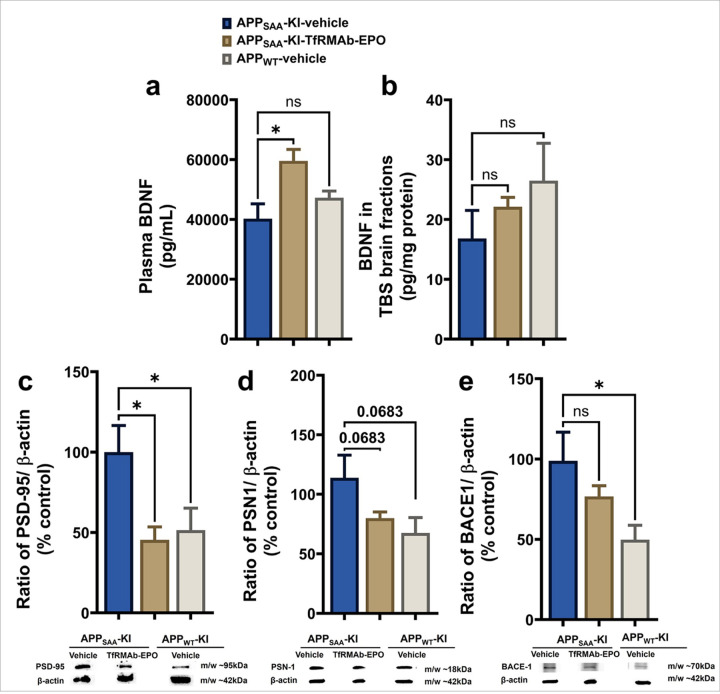
Effect of chronic TfRMAb-EPO treatment on the brain-derived neurotrophic factor (BDNF) and the proteins involved in Aβ production and post-synaptic health. TfRMAb-EPO treatment significantly increased the plasma BDNF levels (a), with no change in the brain BDNF levels in APP_SAA_ KI mice compared to the vehicle-treated APP_SAA_ KI mice. A significant decrease was observed in the protein levels of PSD-95 (c), with a trend of decrease in PSN1 expression levels (d) between the vehicle-treated and TfRMAb-EPO-treated APP_SAA_ KI mice. No change was observed in the expression levels of BACE1 (e) between vehicle-treated and TfRMAb-EPO-treated APP_SAA_ KI mice. Full-blot images are shown in Supplemental Figure. 2. Data are presented as mean ± SEM of n = 4–6 per group and were analyzed using the one-way ANOVA with Holm-Sidak’s post-test. *p<0.05. ns: non-significant.

TfRMAb-EPO treatment significantly increased plasma BDNF levels in APP_SAA_ KI mice compared to vehicle-treated APP_SAA_ KI mice (p<0.05, Fig. 8a). No significant change in the plasma BDNF levels was observed with vehicle-treated APP_WT_ mice compared to vehicle-treated APP_SAA_ KI mice (Fig. 8a). Though a trend toward an increase was observed, TfRMAb-EPO treatment did not significantly increase BDNF levels in the TBS-soluble brain fractions compared with the vehicle-treated APP_SAA_ KI or APP_WT_ mice (Fig. 8b). Further, we investigated the effect of chronic TfRMAb-EPO treatment on the levels of proteins involved in post-synaptic health, Aβ production, and BBB integrity. A significant downregulation in the expression of the post-synaptic protein, PSD-95 (p<0.05, Fig. 8c), was observed in the brains of TfRMAb-EPO-treated APPSAA KI mice and vehicle-treated APPWT mice compared to vehicle-treated APPSAA KI mice. TfRMAb-EPO treatment showed a strong trend toward a downregulation of PSN1 (p=0.068, Fig. 8d) in the brains of APP_SAA_ KI mice compared to vehicle-treated APP_SAA_ KI mice, similar to PSN1 expression in the vehicle-treated APP_WT_ mice. The expression levels of BACE1 (Fig. 8e) were not significantly altered by the chronic TfRMAb-EPO treatment in the APP_SAA_ KI mice, but BACE1 expression was significantly lower in the vehicle-treated APP_WT_ mice compared to vehicle-treated APP_SAA_ KI mice (Fig. 8e). The full western blot images are shown in Supplemental Figure 2.

## Discussion

The overall aim of this study was to determine the dose of the brain-penetrating modified EPO analog (TfRMAb-EPO) that would result in the most favorable pharmacokinetic and safety profile for the treatment of AD mice. Following a single SQ dose, increasing doses of TfRMAb-EPO resulted in increased plasma exposure. However, long-term repeated SQ dosing for four weeks was associated with a significant reduction in plasma exposure from doses ranging between 3–20 mg/kg, with no changes observed at lower doses. These findings are consistent with the previous studies showing a reduction in plasma concentrations following chronic dosing of TfRMAb alone or attached to a therapeutic moiety [11, 20–22]. The reduction in plasma concentrations was observed within one week of TfRMAb-EPO dosing and appeared to decrease with an increase in the injected dose, such that the reduction was more pronounced at the 3 mg/kg chronic dose compared to the 20 mg/kg chronic dose of the modified EPO. Based on previous work across different species, lower doses (1–2 mg/kg) of TfRMAb-based fusion proteins did not alter plasma pharmacokinetics or brain uptake [23, 24], mid doses between 3–6 mg/kg decreased plasma exposure [11, 20], and higher doses (≥ 20mg/kg) showed a decrease in plasma exposure, albeit to a lesser extent [21, 22, 25]. Therefore, previous work and current results suggest that the relationship between a reduction in plasma exposure and dose of TfRMAb-fusion proteins may be bell-shaped. This reduction in plasma exposure of TfRMAb-EPO was partly restored but continued to be significantly reduced even after the eight-week washout period.

To investigate the potential mechanisms of reduced plasma exposure after repeated dosing, we studied the involvement of tissue sequestration, TfR expression, ADA formation, and EPOR expression. Following acute dosing, organ uptake of TfRMAb-EPO showed a significant positive correlation with plasma TfRMAb-EPO exposure for the liver and brain, and not for the spleen and kidney, most likely due to TfR saturation in the spleen and kidney due to high plasma exposure following acute dosing. Following chronic dosing, we did not see a dose-dependent increase in tissue accumulation of TfRMAb-EPO, suggesting that the reduced plasma exposure of TfRMAb-EPO following chronic dosing is not mediated by an increase in tissue sequestration. In fact, our data showed a significant positive correlation between TfRMAb-EPO organ concentrations and plasma exposure for all organs, suggesting that the plasma concentrations are driving tissue concentrations, and not vice versa, following chronic dosing. Marked reduction in plasma concentrations at 3 mg/kg and 6 mg/kg doses of TfRMAb-EPO following chronic dosing also reduced brain tissue concentrations, which is likely to affect therapeutic efficacy. Some TfRMAb-EPO accumulation was observed in the brain following the 20 mg/kg chronic dosing, while the brain concentrations did not differ significantly between acute and chronic dosing for the 1 mg/kg dose. It can be argued that the lower tissue concentrations of TfRMAb-EPO after 3 mg/kg and 6 mg/kg chronic dosing are not driven by the lower plasma concentrations but are due to saturation of tissue TfR or degradation of TfRMAb-EPO within the tissue. However, this is unlikely given that the tissue concentrations are the highest for the 20 mg/kg dose when the plasma concentrations were also highest.

The lowest dose (1 mg/kg) of TfRMAb-EPO did not alter TfR expression in plasma, kidney, liver, or spleen. Mid doses of 3 mg/kg and 6 mg/kg increased TfR expression in the liver and kidney, respectively, with the most robust effect seen in the spleen. The highest dose of 20 mg/kg significantly increased TfR levels in plasma, kidney, liver, and spleen. These results are consistent with previous work showing an increase in splenic TfR expression with TfRMAb dosing [11], but contrast with work showing a decrease in TfR expression with TfRMAb-fusion proteins [26, 27]. It is possible that the presence of the fusion partner or variations in the antibody format (affinity, species selectivity) or doses have a differential impact on tissue TfR levels. This increase in TfR levels with the 3–20 mg/kg doses may contribute to increased tissue uptake and thereby reduced plasma exposure of TfRMAb-fusion proteins, including TfRMAb-EPO. In the present study, this appears unlikely since we did not see an increase in splenic uptake with the 3 mg/kg and 6 mg/kg doses. Notably, the increase in organ TfR levels after chronic TfRMAb-EPO dosing was reversible, such that TfR levels returned to normal levels following an eight-week washout. Plasma TfR levels continued to be elevated after 20 mg/kg chronic dosing, followed by an eight-week washout, and could potentially impact TfRMAb-EPO bioavailability.

We used a sandwich ELISA to measure the ADA against TfRMAb-EPO. ADA was significantly elevated at 6 mg/kg and 20 mg/kg doses compared with the 1 mg/kg and 3 mg/kg doses at one and two weeks following chronic dosing. Therefore, while ADA may be contributing to reduced plasma exposure at the 6 mg/kg and 20 mg/kg doses, which has been shown for other TfRMAb-fusion proteins [20], the reduced plasma exposure at the 3 mg/kg dose does not appear to be ADA-related. The latter is also consistent with previous work showing low titer ADA at the 3 mg/kg dose of TfRMAb-fusion proteins [21, 28]. Finally, we did not see an increase in tissue EPOR expression with chronic dosing, suggesting that increased EPOR tissue expression does not contribute to increased tissue uptake and clearance of TfRMAb-EPO after chronic dosing (Supplemental Fig. 3).

Specific to the TfRMAb-EPO fusion protein are changes in hematologic indices associated with chronic EPO dosing, which can result in significant hematologic adverse effects [5, 6], specifically after long-term treatment. We found that a single dose of TfRMAb-EPO did not elevate hematocrit at any of the doses (1–20 mg/kg), and the same observation was made following four weeks of chronic dosing at 1–6 mg/kg doses. Notably, EPO alone results in a significant increase in hematocrit at lower or comparable doses [8, 29]. There was a 10% reduction in hematocrit with the 20 mg/kg repeated dosing for four weeks, which was reversible following the eight-week washout. The reduction in the hematocrit with the TfRMAb-EPO is most likely driven by the TfRMAb domain, which is associated with the loss of reticulocytes mediated via the binding of TfRMAb to the TfR-rich reticulocytes, followed by immune-mediated clearance [30].

We saw a dose-dependent increase in spleen weights (splenomegaly) following chronic TfRMAb-EPO dosing at doses between 3–20 mg/kg, paralleling the increase in splenic TfR at these doses. No changes were seen following acute or chronic 1 mg/kg dosing. These findings are consistent with previous work showing spleen enlargement with chronic TfRMAb dosing (without a fusion partner) [11]. One potential mechanism underlying splenomegaly may be increased clearance of TfR-rich reticulocytes and resultant splenic-iron overload. As seen with the hematocrit and TfR expression levels, splenomegaly was reversible and normalized after dosing was stopped. Similarly, changes in the comprehensive plasma diagnostic panel, following the 20 mg/kg chronic dose, were normalized with the eight-week washout (Supplemental Fig. 4).

Based on the results from the pharmacokinetic and safety studies, the 1 mg/kg dose was used to treat the APP_SAA_ KI mice. This dose resulted in stable plasma and brain exposure without greatly altering TfR expression levels, hematocrit, and organ weights. APP_SAA_ KI mice showed a trend toward a decrease in locomotion and no cognitive deficits compared to the APP_WT_ mice at the time of sacrifice, which is consistent with the motor and cognitive changes reported in these mice at 9 months of age [31]. TfRMAb-EPO treatment did not alter locomotion indices of the APP_SAA_ KI mice. Despite the absence of deficits in the spatial reference memory using the Y-maze in the vehicle-treated APP_SAA_ KI mice, TfRMAb-EPO-treated APP_SAA_ KI mice showed a higher preference for the novel arm following fourteen weeks of chronic SQ dosing, indicating increased preference for the novel arm with TfRMAb-EPO treatment. As expected, no 6E10-positive Aβ deposits were seen in the APP_WT_ mice compared to APP_SAA_ KI mice that showed robust 6E10-positive Aβ deposits. Notably, TfRMAb-EPO treatment reduced total brain Aβ load by > 70%. An ELISA for aggregated human Aβ confirmed the reduction in insoluble aggregated human Aβ with TfRMAb-EPO treatment. We have previously shown a reduction in Aβ load with a 3 mg/kg IP or SQ dose of TfRMAb-EPO in the APP/PS1 mouse model of AD [8, 9]. However, TfRMAb-EPO dosing resulted in a 30–40% reduction in Aβ load, which, though significant and in line with the reduction seen with murine analog of Lecanemab in AD mouse models [32, 33], is modest compared to the remarkable > 70% reduction seen in the current study. The dampened therapeutic effect with the 3 mg/kg TfRMAb-EPO dose in the prior studies compared to the current study can now be explained by the significant reduction in the plasma and brain concentrations at the 3 mg/kg repeated dose, which was not observed at the 1 mg/kg chronic dose used in the current study.

EPO modulates key events involved in AD pathogenesis, including Aβ load, tau hyperphosphorylation, and endogenous neuroprotective and neuroregenerative pathways [34]. To elucidate the mechanisms involved in Aβ reduction and cognitive improvement with TfRMAb-EPO in the APP_SAA_ KI mice, we focused on three key pathways: Aβ generation, neuroprotection, and neuroinflammation. EPO can increase the levels of BDNF [35], a key neurotrophin involved in neurogenesis, synaptic function, and maintenance of neuronal plasticity, which is crucial for learning and memory [36]. Reduced BDNF levels have been reported in the CNS and periphery of AD patients [37], and plasma BDNF levels are negatively correlated with brain amyloidosis [38]. Additionally, plasma BDNF levels have been suggested to be associated with neuronal plasticity [39]. In the current study, chronic low-dose treatment of the APP_SAA_ KI mice with TfRMAb-EPO significantly increased the levels of BDNF in the plasma and may underlie the improved performance of the TfRMAb-EPO-treated APP_SAA_ KI mice in the Y-maze test. We also measured the levels of PSD-95 as a marker of post-synaptic health. Though PSD-95 expression is largely shown to be reduced in the AD brain [40], reflecting postsynaptic degeneration, literature shows that PSD-95 expression may vary with disease stage in AD. Some studies have reported an increase in PSD95 levels in the AD brain [41, 42], indicating an increase in excitatory synapses and seizure susceptibility in AD mice, which were restored with PSD-95 suppression [41]. In the current study, PSD-95 levels were significantly elevated in the APP_SAA_ KI brains compared to the APP_WT_ brains, and TfRMAb-EPO treatment of APP_SAA_ KI mice restored PSD-95 expression back to WT values. These results suggest that TfRMAb-EPO treatment restores postsynaptic changes to normal levels in the APP_SAA_ KI mice.

We studied the expression levels of two proteins, PSN1 and BACE1, involved in the amyloidogenic cleavage of APP, leading to Aβ generation [43]. BACE1 expression was significantly elevated in the APP_SAA_ KI brains compared to APP_WT_ mice, with a similar trend in the PSN1 expression, marking an increase in APP cleavage in the APP_SAA_ KI mice. TfRMAb-EPO treatment showed a trend toward a reduction in these key proteins involved in Aβ production, suggesting some modulation of APP cleavage, which is consistent with prior work in humans where recombinant EPO reduced PSN1 and BACE1 in platelets; the latter contain the majority of circulating APP [44]. TfRMAb-EPO did not alter neuroinflammation or BBB tight-junction protein expression (Supplemental Fig. 5), mechanisms that are also significantly implicated in AD pathogenesis [45].

There are some limitations to the presented work. First, the organ-based endpoints (e.g., TfR expression, tissue uptake) are based on a single terminal time point (24 hours) and therefore do not capture the temporal dynamics of these endpoints. Second, sex differences were not studied, and future work in female mice will determine if changes in TfRMAb-EPO PK or efficacy after chronic dosing show sex-dependent changes. Third, though the APP_SAA_ KI mice overcome the issues associated with human APP overexpression in other AD mice, APP_SAA_ KI mice do not show overt cognitive dysfunction despite significant Aβ plaque pathology at the age studied herein. Our sample size was based on neuropathological outcomes (Aβ) rather than behavioral endpoints, which usually require a larger sample size. Future work in older APP_SAA_ KI mice with behavior deficits will be needed to determine if the robust reduction in Aβ pathology translates into cognitive improvements.

## Conclusions

**Figure 9:**
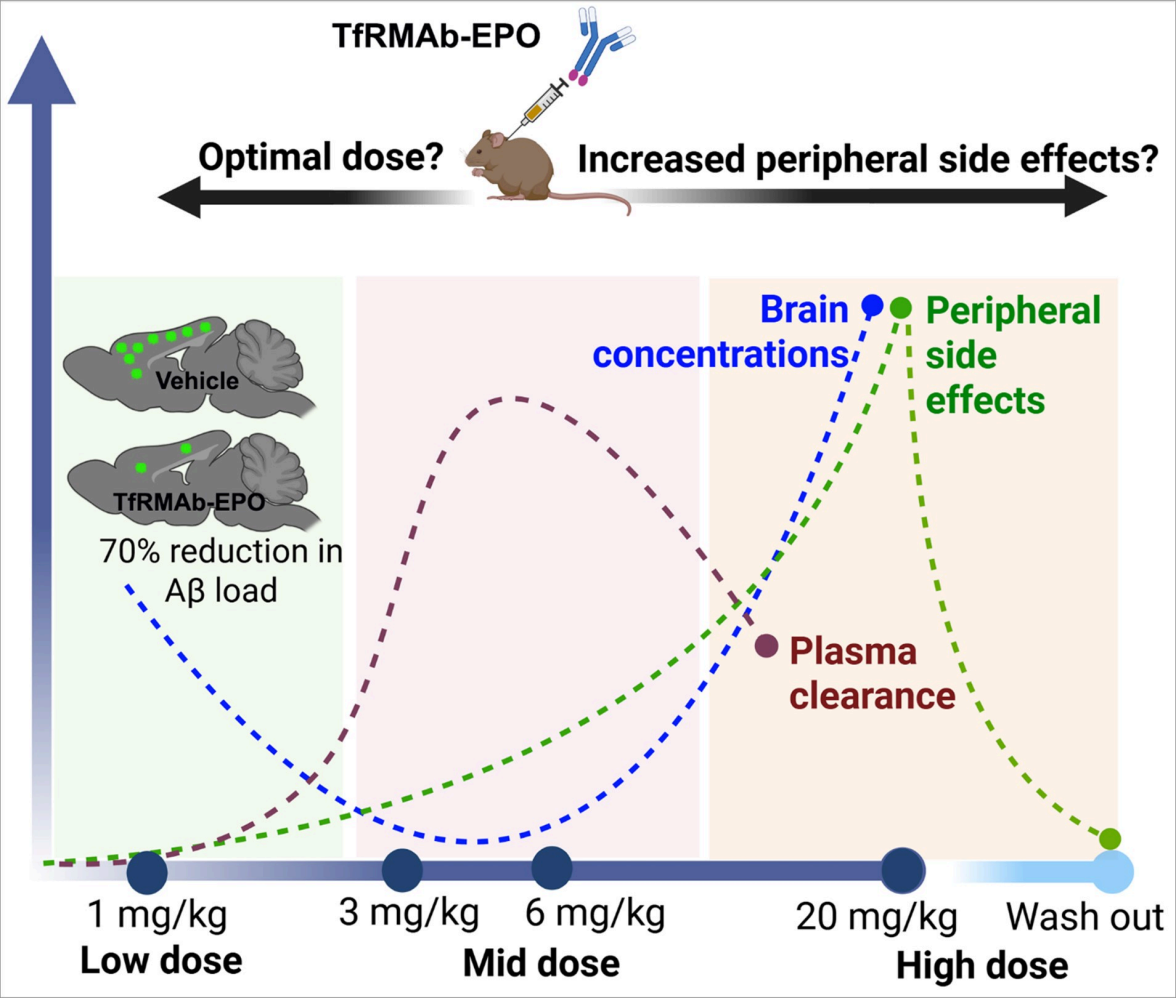
Schematic overview of the main results showing dose-dependent pharmacokinetics and safety of TfRMAb-EPO. Plasma clearance exhibits a bell-shaped relationship with dose, peaking at mid-range doses (3 and 6 mg/kg). In contrast, brain concentrations follow a U-shaped curve, inversely mirroring plasma clearance—lower brain levels are observed when plasma clearance is highest. Peripheral adverse effects increase with the administered dose; however, these effects are largely reversible and tend to resolve upon cessation of treatment. These data suggest that low-dose TfRMAb-EPO is optimal for long-term dosing of AD mice, and our data in APP_SAA_ KI mice show robust reduction in brain Aβ load with low-dose TfRMAb-EPO.

Overall, this study investigated the plasma PK and tissue biodistribution of TfRMAb-EPO, a brain-penetrant EPO analog, across low (1 mg/kg), mid (3–6 mg/kg), and high (20 mg/kg) dosing regimens. Chronic administration revealed a dose-dependent decline in both plasma and brain exposure at mid-doses. Mechanistic evaluation of four potential contributors—tissue sequestration, TfR overexpression, ADA formation, and EPOR overexpression—identified TfR expression and ADA formation as partial contributors to reduced plasma exposure, suggesting multimodal mechanisms may govern TfRMAb-based drug distribution. However, these factors alone do not fully account for the pronounced plasma exposure decrease at mid and high doses. Changes in hematocrit, plasma metabolic indices, splenomegaly, and TfR expression, observed with the high-dose of TfRMAb-EPO were reversible (Fig. 9). These findings are critical for optimizing dosing strategies in longitudinal studies of TfRMAb-based therapeutics, particularly TfRMAb-EPO, given the movement of TfRMAb-based therapeutics into clinical trials for AD (NCT04639050). Our work underscores that less is more, as evidenced by the robust reduction in Aβ load with the low-dose TfRMAb-EPO in the APP_SAA_ KI AD mouse model. Future studies exploring additional mechanisms influencing plasma exposure following repeated dosing of TfRMAb-based therapeutics will further refine dosing and enhance therapeutic efficacy.

## Supplementary Material

Supplementary Files

This is a list of supplementary files associated with this preprint. Click to download.

- Supplementaryfile06.11.25.docx

## Data Availability

The datasets for the current study are available from the corresponding authors upon reasonable request.

## Abbreviations

AD: Alzheimer’s disease
Aβ: Amyloid beta
APP: Amyloid precursor protein
ANOVA: Analysis of variance
ADA: Anti-drug antibodies
AUC: Area under the curve
BACE1: Beta-site amyloid precursor protein cleaving enzyme 1
BCA: Bicinchoninic acid
BBB: Blood-brain barrier
BSA: Bovine serum albumin
BDNF: Brain-derived neurotrophic factor
CHO-K1: Chinese hamster ovary
EPOR: Erythropoietin Receptor
ELISA: Enzyme-Linked Immunosorbent Assay
GAM-AP: Goat anti-mouse light chain (kappa) antibody conjugated to alkaline phosphatase
Gu-HCl: Guanidine hydrochloride
hAβ: Human Aβ
IACUC: Institutional Animal Care and Use Committee
IP: Intraperitoneal
Iba1: Ionized calcium-binding adaptor molecule-1
MSD: Meso Scale Discovery
OD: Optical density
PFA: Paraformaldehyde
PK: Pharmacokinetics
PBS: Phosphate-buffered saline
PNPP: p-Nitrophenyl Phosphate
PVDF: Polyvinylidene difluoride
PSD-95: Postsynaptic density protein 95
PSN1: Presenilin-1
RIPA: Radioimmunoprecipitation assay buffer
RT: Room temperature
SNR: Signal-to-noise ratio
SA-AP: Streptavidin-alkaline phosphatase
SQ: Subcutaneous
SAA: Swedish, Arctic, and Austrian
TfRMAb: Transferrin receptor monoclonal antibody
TREM2: Triggering receptor expressed on myeloid cells-2
TBS: Tris-buffered saline
TBSB: Tris-buffered saline containing 0.1% bovine serum albumin
TBST: Tris-buffered saline with 0.05% Tween-20
TPER: Tissue Protein Extraction Reagent
WT: Wild-type
ZO-1: Zonula occludens-1
